# Retrospective analysis of risk factors associated with Kawasaki disease in China

**DOI:** 10.18632/oncotarget.17530

**Published:** 2017-04-29

**Authors:** Lihua Bai, Tienan Feng, Lifang Yang, Yi Zhang, Xuejuan Jiang, Jiayao Liao, Lihua Chen, Xiaoyan Feng, Yanming Rong, Yuehua Li, Zhiqiang Qin, Jing Qiao

**Affiliations:** ^1^ Department of Pediatrics, Research Center for Translational Medicine, Shanghai East Hospital, Tongji University, Shanghai 200123, China; ^2^ Hongqiao International Institute of Medicine, Shanghai Tongren Hospital and Faculty of Public Health, Shanghai Jiao Tong University School of Medicine, Shanghai 200025, China; ^3^ Shanxi Provincial People's Hospital, Xi’An Jiaotong University, Xi’an 710068, China; ^4^ The 3rd Clinical Medical School, Three Gorges University, Yichang 443002, China; ^5^ Departments of Genetics, Louisiana State University Health Sciences Center, Louisiana Cancer Research Center, New Orleans, LA 70112, USA

**Keywords:** Kawasaki disease (KD), typical KD, incomplete KD, IVIG ineffectiveness, retrospective analysis

## Abstract

In order to provide early intervention for coronary artery lesion (CAL) caused by Kawasaki Disease (KD), we analyzed clinical characteristics of typical and incomplete KD cases from 1998 to 2008 in Northwest and Central China. A total of 383 patients included 298 cases of typical KD and 85 cases of incomplete KD. The morbidity of incomplete KD was 28.5%, a percentage significantly lower than that of typical KD. The occurrence of bulbar conjunctiva congestion, erythra, crissum red, film-like decrustation, lip red, rhagades, raspberry tongue, bilateral toe-end decrustation, limb sclerosis, cervical lymph nodes enlargement, agitation and irritability in incomplete KD group was lower than that in the group of typical KD (*p* < 0.05); however, the occurrence of unilateral toe-end decrustation, scar reappearance erythema, malaise, fatigue, liver incidence was significant higher in incomplete KD group (*p* < 0.05). Based on lab assays and inspection index comparisons, the incomplete KD cases whose C-reactive protein (CRP) and erythrocyte sedimentation rate (ESR) were significantly increased, had significantly higher reduction in blood platelet (PLT). Interestingly, the KD patients with CPR higher than 30 mg/L, ESR higher than 40 mm/h, hepatomegaly and IVIG ineffectiveness, had higher incidence of CAL development. Altogether, our data have indicated differential clinical characteristics between incomplete KD and typical KD, and have identified several high risk factors of KD for CAL, such as hepatomegaly.

## INTRODUCTION

Kawasaki Disease (KD) is a condition that causes inflammation in the walls of medium-sized arteries throughout the body. While the disease occurs in early childhood, it is thought to cause dysfunction of the vasculature system and coronary artery lesions (CAL) later in life. The morbidity rate of KD increased in recent years. Since the first case reported by Dr. Kawasaki in 1967, deep changes of the cardiovascular system associated with KD have also gained attention in clinic [1]. Most of KD cases present the 6 typical symptoms (see below Methods for details), however, some cases that don't completely present with all the typical symptoms are defined as incomplete KD. Those cases are often accompanied to coronary artery dysfunction and cannot be explained with other diseases. The morbidity of incomplete KD in all KD cases is around 10%˜36% and they do not respond well to intravenous immunoglobulin (IVIG) treatment [2–4]. To determine the likelihood of potential adulthood acute coronary artery events, we have performed the retrospective analysis of 383 KD cases with clinical characteristics, treatment and their prognosis collected from 1998 to 2008 in Northwest and Central China.

## RESULTS

### Comparison of the clinical characteristics between incomplete KD and typical KD cases

Among the total 383 patients, there were 85 incomplete KD cases that included 51 males and 34 females (M-F ratio was 1.71:1); 298 typical KD cases included 196 males and 102 females (M-F ratio was 1.92:1). Although slightly different among the two groups, the gender had no statistical significance (*p* > 0.05) (Table 1). In the 85 incomplete KD cases, all the patients had fever for more than 5 days, and the other major clinical characteristics included agitation and irritability (65/85, 77%), bulbar conjunctiva congestion (63/85, 74%), lip red, rhagades and raspberry tongue (46/85, 54%), crissum red and film-like decrustation (44/85, 52%); 42 cases had erythra (42/85, 49%), bilateral toe-end decrustation (34/85, 40%), hepatomegaly (32/85, 38%), limb sclerosis (31/85, 36%). Taken together, the occurrence rates of unilateral toe-end decrustation, scar reappeared erythema, sag, lassitude and hepatomegaly in incomplete KD cases were significantly higher than in those of typical KD cases. The occurrence rates of bulbar conjunctiva congestion, erythra, crissum red, film-like decrustation, lip red, rhagades, raspberry tongue, bilateral toe-end decrustation, limb sclerosis, cervical lymph nodes enlargement, agitation and irritability in incomplete KD cases were obviously lower than those in typical KD cases. Nevertheless, the occurrence rates of pyrexia, crissum hyperaemia desquamation and stomachache had no statistical difference between these two groups.

**Table 1:** Comparison of the clinical characteristics between incomplete KD and typical KD cases

		KD condition		Chi-square	*p* value
	0 (negative)1 (positive)	Incomplete	Typical		
Hyperemia bulbar	0	22	15	**32.64**	< 0.001*
	1	63	283		
erythra	0	43	35	**61.53**	< 0.001*
	1	42	263		
Peeling of red crissum membrane	0	41	161	0.89	0.35
	1	44	137		
Lip red, rhagades and raspberry tongue	0	39	54	**27.72**	< 0.001*
	1	46	244		
Bilateral toe-end decrustation	0	51	68	**42.69**	< 0.001*
	1	34	230		
Unilateral toe-end decrustation	0	66	291	**41.82**	< 0.001*
	1	19	7		
Limb sclerosis	0	54	113	**17.64**	< 0.001*
	1	31	185		
Cervical lymph nodes enlargement	0	55	74	**47.08**	< 0.001*
	1	30	224		
Scar reappeared erythema	0	65	256	4.34	0.037*
	1	20	42		
Agitation, irritability	0	20	32	9.22	0.002*
	1	65	266		
Sag, lassitude	0	67	264	5.37	0.02*
	1	18	34		
Stomachache	0	55	224	3.66	0.056
	1	30	74		
Hepatomegaly	0	53	247	**16.43**	< 0.001*
	1	32	51		
WBC increase ≥ 15 × 10^9^/L	0	8	20	0.071	0.4
	1	77	278		
HB decrease ≤ 10 g	0	26	62	3.58	0.059
	1	59	236		
PLT increase ≥ 450 × 10^9^/L	0	11	17	5.11	0.024*
	1	74	281		
CRP increase (≥ 30 mg/L)	0	3	63	**14.38**	< 0.001*
	1	82	235		
ESR acceleration (≥ 40 mm/h)	0	0	94	**35.53**	< 0.001*
	1	85	204		
Coronary artery dilating	0	62	220	0.027	0.87
	1	23	78		
Gallbladder enlargement	0	74	270	0.91	0.34
	1	11	28		
Chest radiography abnormality	0	59	197	0.33	0.56
	1	26	101		
IVIG-ineffectiveness	0	76	292	**12.92**	< 0.001*
	1	9	6		

Comparing the results of laboratory tests and ultrasound data between these two groups, the occurrence rate of C-reactive protein (CRP) increase was 96.5% and Erythrocyte sedimentation rate (ESR) acceleration was 100% for incomplete KD cases, which were significantly higher than the values observed in typical KD cases (*p* < 0.05). The occurrence rate of blood platelet (PLT) increase was 87.1% for incomplete KD cases, which was significantly lower than that of typical KD cases (94.3%, *p* < 0.05). However, the occurrence rates of white blood cell (WBC) increase, Hemoglobin (Hb) decrease, coronary artery ectasia, gallbladder enlargement and chest radiography abnormality had no statistical difference between these two groups. Finally, IVIG-ineffectiveness probability of incomplete KD cases (10.6%) was significantly higher than that of typical KD cases (2.0%, *p* < 0.05).

### Risk factors assessment comparison between typical KD and incomplete KD cases

For the patients who received IVIG therapy (*n* = 383), incomplete KD patients’ average age was 2.87 ± 2.23 and typical KD patients’ average age was 3.01 ± 2.35. The average age between two groups had no statistical significance difference. CRP over 30 mg/L, ESR over 40 mm/h, Hepatomegaly and IVIG-ineffectiveness were high risk factors of typical KD (Table 2). Children under one of these conditions, CRP over 30 mg/L, ESR over 40 mm/h, hepatomegaly and IVIG-ineffectiveness showed around 10 folds risk to get typical KD. Other indicators including limb sclerosis, cervical lymph nodes enlargement, bilateral toe-end decrustation, bulbar conjunctival congestion, erythra, lip red rhagades and raspberry tongue were also counted as risk factors, and which showed less than 3 folds to get typical KD (*p* < 0.05).

**Table 2:** Risk factors assessment comparison between typical KD cases and incomplete KD cases

	Exp (B)^1^	S.E. ^2^	Sig.^3^
Gender	0.74	0.46	0.54
Bulbar conjunctival congestion	0.05	0.68	< 0.001*
Erythra	0.04	0.58	< 0.001*
Lip red, rhagades and raspberry tongue	0.23	0.55	0.01*
Bilateral toe-end decrustation	0.18	0.55	< 0.001*
Unilateral toe-end decrustation	2.81	1.03	0.32
Limb sclerosis	0.07	0.58	< 0.001*
Cervical lymph nodes enlargement	0.03	0.61	< 0.001*
Hepatomegaly	9.96	0.56	< 0.001*
CRP increase (≥ 30 mg/L)	21.32	1.17	0.01*
ESR acceleration (≥ 40 mm/h)	60.14	1.24	< 0.001*
IVIG-ineffectiveness	14.81	0.84	< 0.001*
Constant	2.90	1.44	0.51

*Set the non-occurrence condition (0) as the comparison object.

^1^Exp(B) represents the OR value between typical KD cases and incomplete KD cases.

^2^S.E. represents the standard error of B.

^3^Sig. represents the statistics significance.

### Evaluation of risk factors associated with CAL

Next, we evaluated risks possibly associated with CAL. As shown in Table 3, the major risk factors include increased CRP (≥ 30 mg/L), ESR acceleration (≥ 40 mm/h), hepatomegaly and IVIG-ineffectiveness. For instance, our results indicate that the risk of CAL in patients with hepatomegaly is about eight times higher than in those without hepatomegaly.

**Table 3:** Risk factors of CAL

	Exp (B)^1^	S.E.^2^	Sig.^3^
KD	1.51	0.35	0.24
Gender	0.86	0.27	0.57
WBC increase ≥ 15 × 10^9^/L	1.54	0.47	0.36
HB low ≤ 10 g	0.90	0.31	0.73
PLT increase ≥ 450 × 10^9^/L	1.83	0.47	0.20
CRP increase (≥ 30 mg/L)	2.66	0.33	< 0.001*
ESR acceleration (≥ 40 mm/h)	3.71	0.30	< 0.001*
Hepatomegaly	8.05	0.62	< 0.001*
Chest radiography abnormality	1.04	0.27	0.89
IVIG-ineffectiveness	0.02	0.65	< 0.001*

*Set the non-occurrence condition (0) as the comparison object.

^1^Exp(B) represents the OR value between typical KD cases and incomplete KD cases.

^2^S.E. represents the standard error of B.

^3^Sig. represents the statistics significance.

**Figure 1:**
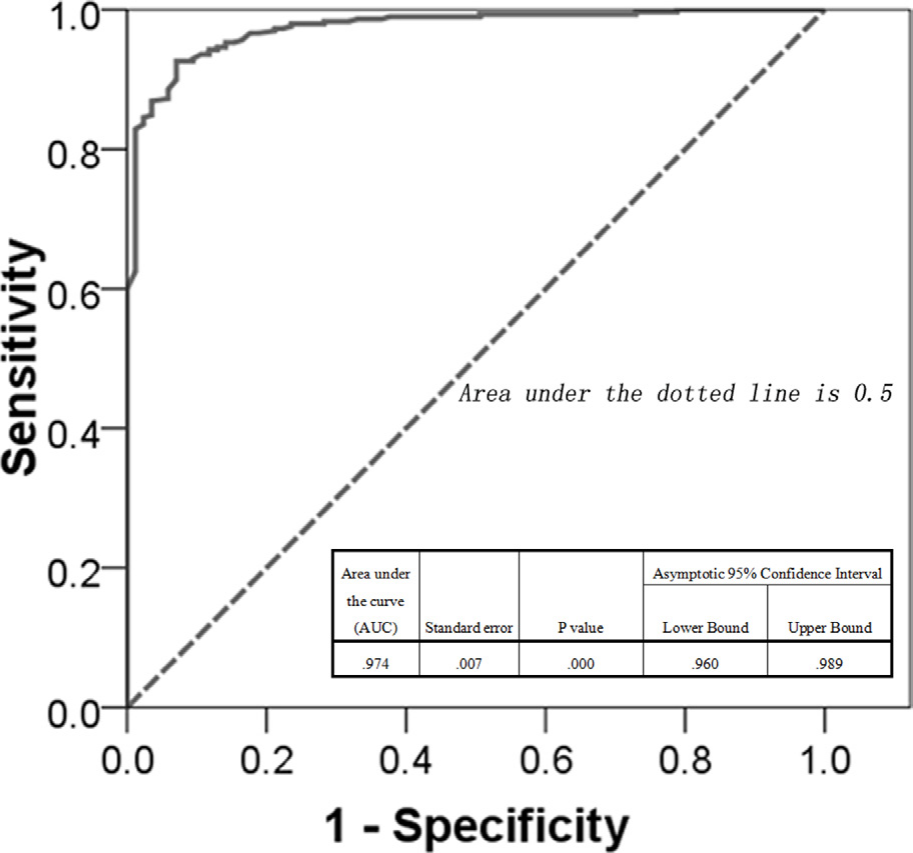
ROC curve of our model (AUC = 0.974) The values calculated by our model were used as the predictors, and the outcome indicators were set as reference measure. The curve was produced by using ROC module of SPSS20.0 software, which illuminating the predicting accuracy of our model.

Based on these factors, we have established a new assessment model. For our clinical samples, the sensitivity was 96.2, the specificity was 83.5 and the accuracy was 93.5. The AUC (area under the curve) score was 0.974 (Figure 1). While this model has potential clinical values, more samples are needed to validate and eventually improve it.

## DISCUSSION

Previous studies have provided the evidences of some epidemiologic features about Chinese KD cases [5–7]. However, to our knowledge, this is the first analysis of the KD hospitalized children in big hospitals in Northwest and Central China. The incidence of coronary artery lesion in incomplete KD cases in our study was around 22.2%, which is consistent with previous data reported oversea by Rowley *et al* (10%˜36%) [8], and domestically by Wang et al (19.4%) [9].

In this study, the occurrence rates of bulbar conjunctiva congestion, erythra, crissum red, film-like decrustation, lip red, rhagades, raspberry tongue, bilateral toe-end decrustation, limb sclerosis, cervical lymph nodes enlargement, agitation and irritability were significantly lower than typical KD. The occurrence rate of cervical lymph nodes enlargement was 35.3%, which also matched previous report [10]. Furthermore, the occurrence rates of fever, crissum congestion desquamation, and stomachache between two groups had no statistical difference. Thus the atypism of incomplete KD's clinical features could be seen.

This research shows CAL occurrence rate in incomplete KD was 27.1% and that in typical KD was 26.2%, which had no statistical significance difference and indicated that all of the patients can erupt simultaneously CAL lesion. Those data were in line with one research in Japan that CAL lesion rate in incomplete KD was not higher than that in typical KD [2], but CAL lesion rate in northwest and central China was higher than that in Japan. Our findings, however, were in disagreement with one inland literature [11] showing that incomplete KD erupts simultaneously in coronary artery lesion more easily than typical KD. From the experimental point of view, the occurrence rates of CRP rising and ESR speed-up were obviously higher in incomplete KD cases compared to typical KD, while blood platelet increase was significantly lower in incomplete KD compared to typical KD. Finally, WBC increase, Hb decrease, CAL, gallbladder enlargement and chest radiography abnormality between the two groups had no statistical difference. At present, most of domestic and overseas researchers showed that the parameters of CRP rising ≥ 30 mg/L and (or) ESR speed-up ≥ 40 mm/h combined with clinic analysis can be considered as diagnostic factors for incomplete KD, and our research confirmed the validity of those factors. In addition, our research showed that IVIG ineffectiveness rate in incomplete KD was 9%, a percentage significantly higher than typical KD. At present, the mechanism of IVIG ineffectiveness is still unclear, so there is no consensus on the treatment strategies for IVIG-ineffective patients. Kohayashi had reported results from a study performed in multi-center, foresight and random research observing severe KD patients (danger grade > 5). The study concluded that the occurrence rate of coronary artery lesion could be reduced by standard regimen of IVIG combined with aspirin and prednisolone [12]. The experimental group had 121 cases and control untreated group had 121 cases. The occurrence rate of coronary artery lesion of experimental group (3%) was lower than that of control group (23%). The fever time of experimental group was shorter, and its IVIG second request was also obviously less than that of standard scheme group (*p* < 0.001).

According to Harada score and related literature, risk factors for CAL lesions in KD are the following; being a male, being under 1 year old, having plasma albumin 35g/L or less, CRP strong positive, WBC > 12 × 10^9^/L, PLT < 300 × 10^9^/L and Hc < 35% [13–15]. Interestingly, this study showed CRP > 30 mg/L or ESR > 40 mm/h, hepatomegaly and IVIG-ineffectiveness were important risk factors for CAL lesions, while gender and increased WBC had no correlation with CAL lesions in KD.

In summary, KD is currently thought to be the main reason for the occurrence of heart diseases in children. It has been reported that KD patients have thrombosis related acute coronary syndrome risk in their young adulthood. In recent years the influence of acute coronary events in adulthood in children who had coronary artery structure and function changed after KD in childhood has received particular attention. To reduce CAL complications and to improve the prognosis it is recommended to monitor and record from childhood to adulthood the parameters of CRP ≥ 30 mg/L, ESR ≥ 40 mm/h, hepatomegaly and IVIG ineffectiveness, as those parameters are associated with the risk of developing heart diseases.

## MATERIALS AND METHODS

### Clinical data

From 1998 to 2008, Shanxi Provincial People's Hospital Attached to Xi’An Jiaotong University in Northwest China had treated 137 KD patients including 39 incomplete KD cases; the Third Clinical Medical School Attached to Three Gorges University in Central China had treated 246 KD patients including 46 incomplete KD cases. Incomplete KD patients’ average age was 2.87 ± 2.23 and typical KD patients’ average age was 3.01 ± 2.35. The patients with symptoms not fully matching KD or incomplete KD diagnostic criteria (see below) or with missing medical records were excluded in our study. We have the access to all the involved patients’ identifying information when collecting the data. All the clinical data for these patients (e.g. specific diagnostic/laboratory tests or clinical observations) were acquired from their individual medical records. All the research involving human participants have been approved by Review Board (IRB) Committee of the institutes mentioned above, and written informed consent have been obtained from the participants.

### Diagnostic criteria

Typical KD has 6 diagnostic criteria [16]: ① Fever for 5 days or above. ② Bilateral bulbar conjunctival congestion. ③ Lip and mouth pathological changes including lip dry, redness and rhagades, mouth and pharyngeal diffuse congestion. ④ Extremities pathological changes including hand-foot swelling, palmar and plantar erythema, finger-toe-end membraniform decrustation in recovery phase. ⑤ Pleomorphic erythra which appears mostly in the trunk but without hydroa and incrustation. ⑥ Lymphonodicervicales’ non-suppurative swelling. Typical KD must meet at least 5 of the 6 criteria listed above. The diagnostic criteria of coronary artery dilation is inner diameter > 3 mm for children under 5 years old and inner diameter > 4 mm for children over 5 years old. In addition, the diagnostic criteria for coronary aneurysm are coronary artery localized or diffused dilation whose diameter is 1.5 times larger than adjacent normal coronary artery. Small coronary aneurysm's inner diameter is set as 4.1 to 4.9 mm, medium coronary aneurysm's inner diameter is 5.0 to 7.9 mm, and large coronary aneurysm's inner diameter is more than 8 mm. Incomplete KD is defined as KD in which less than 5 of the 6 criteria, e.g. only 3 to 4 criteria, and for which other diseases have been excluded. The criteria of IVIG-ineffective KD or IVIG refractory KD is defined as body temperature kept over 38°C for more than 48 hours after IVIG treatment or recrudescent fever occurs 2 to 7 days or even 2 weeks after treatment and has at least one KD's diagnostic criteria [10, 17, 18].

### Therapeutic strategies

After definitive diagnosis, all patients are treated as typical KD, including oral administration of aspirin with the following regimen: a dose of 30˜50 mg/kg 3 times a day for fever disappeared, followed by a dose of 5 mg/kg/d for 6˜8 weeks. If the coronary artery lesions occurred, the aspirin treatment was prolonged until the coronary artery was rehabilitated; in addition, IVIG was administered at the dose of 1˜2 g/kg one or two times a day for 2–3 days.

Statistical analysis was performed using the SPSS20.0 (SPSS, Chicago, IL, USA) software. Measurement data was depicted as x ± SD; two-tailed *t*-test analysis was used for comparison between two groups. The enumeration data were compared by the Chi-square test or the Fisher's test. Possible interactions between KD and co-morbidity factors were evaluated through an unconditional binary logistic regression analysis represented as odds ratio at 95% CI. Statistical significance was set at *p* < 0.05.
